# Esports buffs: the perceived role of fans and fandoms in U.S. collegiate programs

**DOI:** 10.3389/fspor.2024.1410929

**Published:** 2024-09-11

**Authors:** Amanda C. Cote, Md Waseq Ur Rahman, Maxwell Foxman, Andrew Wilson, Brandon C. Harris, Onder Can, Jared C. Hansen

**Affiliations:** ^1^ Department of Media and Information, Michigan State University, East Lansing, MI, United States; ^2^ Esports and Games Research Lab, School of Journalism and Communication, University of Oregon, Eugene, OR, United States; ^3^ Department of Computer Science, Design and Journalism, Creighton University, Omaha, NE, United States; ^4^ School of Journalism and Communication, University of Oregon, Eugene, OR, United States; ^5^ Department of Journalism and Creative Media, University of Alabama, Tuscaloosa, AL, United States; ^6^ School of Communication & Design, RMIT University-Vietnam, Ho Chi Minh City, Vietnam

**Keywords:** collegiate esports, fans, fan labor, free labor, audience commodity, qualitative research, in-depth interviews

## Abstract

**Introduction:**

Collegiate esports—organized competitive gaming—has expanded rapidly in the United States, drawing in student players, broadcasters, and support staff, as well as university employees. Universities have invested financially in esports, hoping to capitalize on gaming fandom to attract prospective students and enhance campus community integration. Little research, however, addresses collegiate esports fandom in depth.

**Methods:**

Drawing on thirty-one in-depth interviews with collegiate esports players, student workers, program directors, and administrators, this article investigates how collegiate esports participants perceive and discuss their fans.

**Results:**

We identify three central themes related to fans in the dataset: discussions of fans’ role in the collegiate esports environment, comparisons between esports and traditional sports fans, and concerns about the underutilization of fans within collegiate esports spaces. Subsequently, we theorize these themes through existing research on professional esports and traditional collegiate sports fandoms, as well as through the concept of “fan labor,” or how the productive work of fans provides value to the nascent industry.

**Discussion:**

This article thus not only specifically explores how collegiate esports programs are normalizing fan labor as an essential part of their practices, but also questions who benefits from this relationship and how. Investigating collegiate esports fans as an under-researched group additionally provides a new perspective on how fan labor integrates with media industries more broadly.

## 1 Introduction

Collegiate esports—campus-based, organized competitive gaming—has expanded rapidly in the United States, with over 200 programs founded countrywide since 2014 (1). Students increasingly participate in collegiate esports as players, broadcasters, and support staff, while university faculty and staff take on administrative and organizational roles. Universities have also invested financially in esports, with some schools (approximately 70% of programs) offering scholarships for esports players while others (potentially as high as 92% of programs) have built esports-specific facilities or competitive arenas (2, 3). Many campuses hope to scale their esports programs by cultivating support from fans and students, as well as sponsors. This support is crucial to distinguishing organized collegiate esports competitions from informal gaming clubs. Collegiate esports thus formally recognizes students’ gaming culture interests, but it is also an emerging target for various stakeholders. For the professional gaming industry, collegiate esports cultivates passionate players or backstage participants; for universities, collegiate esports’ overlap with gaming fandom creates another avenue to attract prospective students and enhance campus and student life.

Though universities, players, and student workers have already invested heavily in collegiate esports programs, there exists little to no research on collegiate esports fans. Research on professional esports recognizes fans’ pivotal role as tournament attendees or spectators, as well as their support of players, teams, and sponsor brands (4, 5). Similarly, studies of traditional collegiate sports suggest that robust fandoms benefit the university in terms of student recruitment and retention, but fans themselves receive benefits such as social capital and sense of community in exchange (6–9). Professional esports and collegiate traditional sports thus rely heavily on invested fans, but it is unclear to what extent this holds true for collegiate esports. This study therefore draws on in-depth interviews with collegiate esports participants and administrators to ask:

1. How do players, program directors, and support staff perceive the role of fans in collegiate esports?
2. How do players, program directors, and support staff perceive the challenges or limits that collegiate esports programs face in developing a robust fan base?

Through these questions, we investigate how fans are understood and positioned by program stakeholders. These individuals’ expectations help structure fan engagement with the collegiate esports ecosystem, setting the stage for where and how fans are invited in. Using a grounded theory approach to ensure our conclusions emerged from our participants’ lived experiences, we first found that interviewees viewed fans as essential to the success of collegiate esports, helping legitimize programs to university administrators and directly supporting current and future students. Additionally, participants made several comparisons between esports fans and traditional sports fans, while also expressing concern about the fledgling nature of collegiate esports fandom.

We subsequently theorize these results through the lens of fan labor, or the ways in which fans’ presence and work supports organizations such as collegiate esports programs. Media industries, including games and esports, are increasingly reliant on fans to not only purchase and support specific products, but also to serve as content creators, community builders or moderators, word-of-mouth marketers, and more (10, 11). Based on our interviews, we find that collegiate esports similarly relies heavily on fans to support, justify, and expand the field. Indeed, given their novelty status, collegiate esports programs may be particularly invested in fan labor as it helps elevate them from informal clubs to official varsity status, while also building brand value for the university. This raises the concern that unpaid or exploitative labor practices could come to be built into the collegiate esports environment. Simultaneously, however, interviewees suggest that both fans and program participants receive intangible benefits from their involvement in esports, indicating the potential for symbiosis between programs, universities, and fans. We investigate these claims not only to show which practices and beliefs are normalizing within collegiate esports but also to provide a critical perspective on the various forms of labor that undergird these practices. The article will thus first discuss the concept of fan labor in more detail before investigating its specific role in professional esports, collegiate sports, and finally, the specific field of collegiate esports.

## 2 Conceptual framework

Popular perceptions of esports fandom and spectatorship often consider these activities as leisure (meaning non-work) activities (12). Such a perspective, however, neglects the many connections these activities have to capital, economic opportunities, and labor [as leisure studies scholars have long contended, e.g., (13)]. Time and energy spent on esports is often, for instance, done in pursuit of prize money, scholarships, or professional careers. Spectators and fans also generate value through both direct (e.g., buying merchandise or event tickets) and indirect [e.g., as an audience commodity, marketed to advertisers and sponsors; (14)] investment in games, players or teams, and the overall esports industry. Esports on college campuses are no less connected to flows of capital, as esports programs are used to recruit and retain students (and their tuition dollars) (15, 16). We therefore position this analysis using the conceptual lenses of free labor (17), fan labor (10), and the audience commodity (14) to account for the important role fans play in media industries overall and esports specifically.

Free labor, originally proposed by Terranova (17), describes work that is “simultaneously voluntarily given and unwaged, enjoyed and exploited” (p. 34). She theorized this concept via the common early Internet practice of community members providing value, information, and creative content for a company or platform without expectation of pay. Terranova positions free labor at the heart of platforms like the Internet, where one needs continuous updates “to maintain interest in it and fight off obsolescence” (p. 48). Free labor becomes a means for companies to complete these updates and remain relevant at low costs. As Terranova points out, “in 1996 at the peak of the volunteer moment, over thirty thousand ‘community leaders’ were helping AOL to generate at least $7 million a month” (49), showing how labor conducted for free still produces significant monetary value. Moreover, free labor builds non-material outcomes, like communities and social ties, at the same time as it produces material ones like profit. Terranova is quick to note that free labor “is not necessarily exploited labor” (48), as it can be exchanged for non-material yet meaningful forms of value.

Fan studies scholar Abigail De Kosnik (10) proposed fan labor as a sub-category of free labor. She writes,

*Online fan productions constitute unauthorized marketing for a wide variety of commodities—almost every kind of product has attracted a fandom of some kind. […] Fan activity, instead of being dismissed as insignificant and a waste of time at best and pathological at worst, should be valued as a new form of publicity and advertising, authored by volunteers, that corporations badly need in an era of market fragmentation. In other words, fan production is a category of work. (99)*

As fans produce materials—art, fiction, trailers, livestreams, etc.—around a medium or product of interest, they promote and draw attention to that product, providing free advertising. Additionally, they build social and community value into the products of which they are fans, providing spaces and incentive to share enthusiasm and information. “Perhaps most importantly, fan work creates fan community—fandom itself—through the production and maintenance of affective ties” [(18), p. 78]. As with Terranova's overall concept of free labor, fan labor produces both material and non-material outcomes for companies, but also for fans themselves.^1^

Finally, as media consumers, fans fulfill indirect economic roles as an audience commodity. Elaborated by political economists such as Dallas Smythe (14) and Eileen Meehan (21), the audience commodity describes how media consumers’ leisure time becomes a form of labor under systems of advertising. Although consumers may not pay directly for media like broadcast television or livestreams, the presence of ads means (1) that money has been exchanged between the media company and the advertiser and (2) that the viewer has been “sold.” As advertisers aim to reach their desired audience, they purchase consumer attention by buying ad slots in their target market. While free labor and fan labor thus describe many of the deliberate practices and actions fans take, the concept of the audience commodity accounts for their often-unconscious incorporation into systems of exchange via exposure to advertisements and sponsorships.

Despite these clear connections between fandom and economic value, fan labor often is performed outside of the traditional boundaries of work. As Stanfill and Condis (11) put it, “Fans freely engage in these activities—or they are at least not coerced by the intractable need to earn a living. People enjoy doing them. Thus, it *seems* as if it isn't really labor” (para. 3.2, emphasis added). While these authors proceed to demonstrate how fan practices *are* labor, they recognize that it can be difficult for fans and stakeholders to view these practices *as* labor. Fans are also often explicitly anticommercial, either to circumvent copyright concerns (10, 18) or because they perceive their fan productions as gifts and fandom as a gift economy, in which the benefit to be gained is social giving and community rather than profit (22, 23). Fans who take this perspective often resist framing their work *as work* and as something that should be paid.

Thus, theorization of fan labor is often split. Some theorists view fan labor as an exploited commodity. McCutcheon and Hitchins (24), for instance, specifically view esports fans as exploited, arguing “without their involvement […] the eSports food chain would find it difficult to exist” (p. 70) and pointing out “just because an activity is fun, does not mean it is not generating surplus economic activity and therefore is not work.” (p. 76). Addressed further below, authors like McCutcheon and Hitchins recognize that esports relies on fan investment and attention to succeed. Without viewers, both competitions and practice events or livestreams would lack excitement and emotionality, and they would fail to draw revenue. Because fans contribute value without pay, they can be viewed as exploited. Other theorists, however, take fans at their word when they view fandom as a gift economy—a system in which materials are freely shared without expectation of direct payment—and feel they receive benefits in exchange for their labor (22). Fan scholar Bertha Chin points out, “rather than merely assuming that fans are exploited by the media industry when collaborating with media producers, it is important to acknowledge their voice in this collaboration, and that there may be other motivations at play” (para. 6.1). Chin and others see fans as building affective communities from which they gain real social, emotional, and—at times—economic benefits.

Here, we attempt to walk a middle ground between these perspectives. We start by recognizing fan labor *as* labor, in that fans create value for collegiate esports teams, the broader industry, and their universities. At the same time, we attempt to consider the (im)material benefits fans may find or produce during this process. In line with Stanfill and Condis (11), “once we have conceptualized fan work as generating value, we can also inquire into how that value is distributed and whether work circulating between fans in gift economies or among fans and industry is potentially exploited labor” (para. 1.2). We frame this positioning further throughout our literature review and analysis.

## 3 Literature review

With collegiate esports’ rapid expansion, research into this ecosystem has grown accordingly [e.g., (25)], addressing an array of questions regarding student esports athletes’ campus role (2), whether esports qualify as intercollegiate sport (26, 27), and how programs engage student labor (28). Research has even discussed how to build programs effectively (29), and how to address concerns about diversity, inclusion, and Title IX, the U.S. law mandating gender equity in educational institutions (30, 31). Collegiate-level fans, however, remain understudied. Therefore, we draw on studies of professional esports fans and of collegiate traditional sports fans to help position our study, and collegiate esports fandom, at the nexus of these areas. More specifically, we collated research on fans’ roles and motivations for participation in these two distinct fields to begin identifying commonalities and points of divergence between professional and collegiate environments, as well as between esports and sports. This lays a foundation from which we can better identify and understand the unique aspects of collegiate esports fandom, specifically, while still situating it with regards to existing communities. We organize discussed literature first by area (esports then traditional collegiate sports) then by topic (fans’ roles then fans’ motivations) to help draw out these main ideas.

### 3.1 The role of fans in professional esports

Studies of professional esports fans show that they serve several different roles within the professional environment. For instance, fans may attend events online or in-person, view livestreams of player training or practices, and support or promote specific players, teams, and organizations (32). Esports fans may be aspiring or amateur competitors themselves, watching current professionals to build skills and knowledge (33). Thus, they also serve as a fount of future players. In this way, fans help support and sustain the professional esports industry, providing value to the game companies and tournament organizers that run it.

Professional esports events, such as tournaments, can draw tens of thousands of in-person spectators, while global online esports spectatorship numbers reach tens or even hundreds of millions of viewers (26, 34). These esports fans contribute their time and energy to attending or viewing events, as well as contributing money to purchase event tickets, subscribing to streaming channels or donating online, and/or buying merchandise (35). Many fans also play the esports game titles they spectate, meaning they spend additional dollars on games themselves and potentially on in-game purchases (36, 37). Finally, fans contribute indirect value to professional esports as “eyeballs” (an audience commodity) for industry sponsors who may have their names, logos, or advertisements on jerseys, posted in game arenas, or in online streams. Esports fans represent a desirable market of “affluent young adults who are passionate about competitive video gaming” (38, p. 525), driving high levels of brand interest and investment on the part of sponsors. Collectively, these various fan roles contribute significant value to the professional esports industry, which is conservatively valued at over one billion dollars (39) although some suggest that its true value is significantly higher (36).

In addition to financial contributions, fans also provide cultural and social benefits to esports events, organizations, and the overall industry. Law and Jarrett (40), for instance, discuss a series of chants (similar to those at professional soccer matches) performed during a *Super Smash Bros* tournament to describe non-commercial esports participation. They argue that these distinctive, localized practices add to the emotional and social experiences of in-person esports events, building unique gaming cultures. Additionally, fans of esports teams often seek out and share information about their favorite teams and players, especially around tournaments (32), which can build social relationships and support for players, ensuring fans are more invested in tournaments and the community.

Positioning the above in terms of fan labor, fans clearly help legitimize esports as an entertainment industry, provide both direct and indirect economic value, and contribute to meaningful social and emotional fan experiences. As Kosnik (10) puts it, “fandom is a form of active production, not passive reception” (p. 99). Other scholars, however, critique the industry's dependence on fan contributions, arguing that fans “are sold to potential advertisers as both the advertising content which encapsulates the product message and as a motivated, self-selecting audiences to receive the message” [(24), p. 70] in ways that devalue their labor.

### 3.2 Esports fan motivations

At the same time, research has uncovered several motivations that drive fans to become and remain involved in esports, suggesting that there are benefits to being esports fans. Studies of motivations for spectating esports, for instance, show that fans generally view esports for elements such as drama, knowledge acquisition, appreciation of player skills, novelty, aesthetics, enjoyment of player aggression, and escapism (33, 41). Being an esports fan also satisfies self-determination needs—competence, autonomy, and relatedness (42). Relatedness has a particularly strong effect on fans’ motivations to spectate esports, “highlighting that the social facets of esports are the most salient drivers of positive emotional attachment.” (ibid, p. 231). Fan motivations vary somewhat between in-person and online contexts, with in-person event attendees enjoying social interaction and player attractiveness at higher rates than online spectators (33). Motivations are also moderated by game genre (34). Despite these small variations, though, the aforementioned studies consistently show a relationship between motivation and self-determination and esports spectatorship. Esports fandom seems to gratify several different individual needs via esports spectatorship and involvement.

Existing research, however, focuses largely on professional esports fans, with less attention paid to their collegiate counterparts. Additionally, in a recent systematic review (43), Rietz and Hallmann found that esports motivations studies “focused on esports spectators as one group instead of looking for differences or clusters” (p. 48). They called for additional nuance in future research, to recognize the diverse segments of esports fans that have arisen as the industry has matured and that may have different relationships to fan labor. In this article, we respond to this call by focusing on collegiate esports programs and the potential roles played by their fans.

### 3.3 Collegiate sports fan roles and motivations

Although collegiate esports fandom has not been addressed in depth, traditional U.S. collegiate athletics (e.g., football, basketball, track and field, etc.) have been studied extensively. Collegiate athletics programs cultivate fandoms that subsequently present several benefits to both universities and fans. Athletic team identification or fandom is, for instance, an important part of student recruitment and retention (44), benefiting the university overall. This is a potential form of fan labor, as athletics fandom feeds back into the university's value. Fans thus serve as both direct supporters of sports teams, players, and the university, as well as indirect brand ambassadors.

Collegiate sports fans also experience benefits from their university athletics programs, which can motivate their participation. Overall sports management research demonstrates a connection between spectatorship and community-building [e.g., (45)], which is also accurate in collegiate contexts (46). Spectatorship of and identification with collegiate sports teams can also bring together diverse student cohorts, contribute to campus social capital (6), improve student psychological health (47), aid in students’ social adjustment on campus (9), and increase perceptions of trust (48) in the student body. The work of fandom again potentially permeates back out to benefit those involved in several non-material ways.

Although there has been little specific attention to collegiate esports, professional esports research shows connections between traditional sports fandom and esports fandom. Cushen, Rife, and Wann (49), for instance, found that “eSport and tSport fans are similar on a majority of measured dimensions, including escape, self-esteem, and group affiliation motivation. These findings suggest that sport fandom is a broad, generalizable construct that applies to both tSports and eSports” (p. 127). There are some differences; traditional sports fans are, for instance, more likely to be fans of overall teams and organizations, while esports fans are more likely to associate with a specific game vs. a team or player (5). Moreover, even those who *do* identify with esports teams tend to identify more highly with traditional sports teams, a result that “speaks to the ubiquity of traditional sports in society” compared to esports’ more emergent nature [(49), p. 137]. Although these findings highlight associations between traditional sports and esports, further research is needed on collegiate esports, specifically, to determine if and how these findings hold up in the campus context.

## 4 Methods

This article emerges from a larger project focused on the growth and institutionalization of collegiate esports programs, which draws on in-depth interviews with collegiate esports players, program directors and administrators, and students who are associated with esports through media outlets, esports venues, or initiatives like graphic design. Interviews address several dimensions of collegiate esports programs, from how they are founded, funded, and located within the university setting, to how they relate to students’ education and career preparation, to how they develop codes of conduct and expectations for participants to follow. Many of these aspects have been previously addressed in separate publications (28, 50–52). For this article, specifically, we focus on moments where interviewees discuss the presence and role of fans within the collegiate esports environment, often in response to questions such as, “How, if at all, has your program built a fandom?”, “Do you have any stories about interacting with fans?”, or, for media production and broadcasting interviewees, “How do you think of fans when broadcasting collegiate esports events?”

We conducted thirty-one in-depth interviews total. Our participants were predominantly young (mean 26 years, median 22, range 19–62), male (77.4%), straight (83.9%), and white (74.2%). They came from nine different U.S.-based programs, meaning this dataset can only speak to U.S. collegiate experiences. Interviews ranged from 60 to 120 min and were semi-structured in format, allowing participants to raise new topics as needed. This approach aimed to let the lived experience of esports participants come through clearly, rather than assuming areas of interest preemptively. Interviews were conducted online, transcribed and cleaned for clarity, then analyzed in the qualitative software Dedoose. One member of the research team coded each interview using a grounded theory approach (53).

Grounded theory is an inductive analytic approach that focuses on generating theory from observed themes within the dataset, rather than from existing hypotheses. It necessitates a systematic process in which the researcher “jointly collects, codes, and analyzes his data and decides what data to collect next and where to find them, in order to develop his theory as it emerges” [(53), p. 45]. We collected an initial set of interviews with a small number of participants, then began data analysis. Interviews were analyzed using a combination of open and axial coding (54), whereby the researcher carefully reads each interview and marks every line or short segment with a code that indicates its meaning, generating new codes as needed to describe the data effectively. Subsequently, the researcher reflects on unit-level codes, possible categorizations, and any overlaps or co-occurrences to determine how they form wider themes or categories. In the case of this article, the researchers first used a “fan” code to mark sections of interview transcripts in which participants discussed fans during open coding. These tagged excerpts then underwent secondary analysis to identify, define, and name themes. Because research on collegiate esports fans is nascent, a grounded theory approach allowed us to first identify *how* collegiate esports participants discussed and framed fans (RQ1). We were then able to return to existing esports and sport fan studies, through the lens of our generated themes, to determine specific areas of interest with which to connect (e.g., fan roles, benefits, and comparisons). Generated themes also drove our choice of conceptual framework, fan labor, as we identified clear overlaps between our dataset and this critical perspective. Similarly, as we investigated challenges to building collegiate esports fandoms (RQ2), we noted that programs often relied on students and existing fans to overcome difficulties, such as onboarding new fans. This again suggested fan labor as a useful framework. Thus, a grounded theory approach allowed us to prioritize participants’ lived experiences while still drawing broader conclusions about fans’ value and motivations to participate within mediated environments.

To ensure consistency between coders, the research team maintained a shared codebook and used an “undecided” code to mark sections of data about which they were uncertain. The team discussed these sections as a group to determine the appropriate coding. Therefore, interrater reliability scores cannot be calculated for this analysis, but they are also not needed due to the research's epistemological and methodological approach, which aims for detailed investigations of a specific group, community, or culture rather than for broad, generalizable findings. Moreover, we view the process of peer debriefing and triangulation between researchers as contributing to the trustworthiness (55) of our study and its methods. We began writing up interview results once a specific code set achieved theoretical saturation, or the point where “no additional data are being found whereby the sociologist can develop properties of the category” [(53), p. 61]. Once we had a clear perspective on the range, definition, and meaning of properties within the relevant category, and found that new codes were no longer emerging, we considered that part of the dataset to be saturated.

We anonymized participants by assigning them a designation from P1 to P31 but list their gender, age, and status (player, student worker, admin) to provide context in our analysis. We opted for these general categories—e.g., combining program directors, student life administrators, and industry workers into one “admin” category—both to diminish participants’ identifiability, and because these individuals tended to share similar perspectives throughout our analysis. See Table 1 for summary participant information.

**Table 1:** Participant demographics.

Gender	Female: 7 (22.6%)^a^
	Male: 24 (77.4%)
Age	Range: 19–62
	Average: 26
	Median: 22
Location	Midwest U.S.: 4 (12.9%)^b^
	Mountain U.S.: 5 (16.1%)
	Northeast U.S.: 3 (9.7%)
	Pacific U.S.: 16 (51.6%)
	South U.S.: 3 (9.7%)
Role	Student gamer/Athlete: 11 (35.5%)
	Student worker: 8 (25.8%)
	Administrator: 12 (38.7%)

^a^ One participant identified as female/non-binary. They are classified as a female in this dataset for the purpose of deidentification.

^b^ One participant is originally from outside the U.S. We have classified them based on current location for the purpose of deidentification.

## 5 Results

Overall, we identified three large themes related to fans in the dataset: discussions of fans’ role in the collegiate esports environment, comparisons between esports fans and ball-and-stick sports fans, and concerns about the underutilization of fans within collegiate esports spaces. As appropriate in a grounded theory approach, these themes were rooted in analysis of the data itself, then theorized through existing literature and the conceptual framework of fan labor. Thus, although we began with two overarching research questions (how esports participants perceive fans and how they discuss challenges), our results are organized into three thematic sections. We view the first two sections as addressing RQ1, while the final section more directly addresses RQ2.

### 5.1 Fans’ role

Players, student workers, and administrators all felt that fans serve important roles within collegiate esports, including: promoting the university brand, legitimizing esports programs, supporting players, and helping build the overall esports industry.

#### 5.1.1 University branding

Participants of all kinds connected collegiate esports programs and fandom to the university's overall publicity and branding, arguing that esports could help universities appeal to younger people and existing esports fans. One esports player, for instance, described collegiate esports programs as a sort of community outreach, stating, “I think it's good for colleges because you're going to get more kids who maybe normally wouldn't come to a school. Maybe they're going to come to the school now because there's an esports program” (P4, 21, Male, Player). Similarly, program directors hosted events and asked players to promote the school on Twitch and social media to strengthen connections between the program and the university's brand to attendees and fans. While in-person events allowed them to bring community members onto the campus, deepening local connections, online events helped them to reach geographically widespread audiences and recruit broadly.

A few participants even mentioned specific student recruitment efforts involving their esports program. One administrator, when asked about relationships with fans, described a “Future [School Mascot]” channel on their esports program's Discord server. They said,

*“Past players, current players, or just anybody who’s a member of the community goes in there and answers questions. […] It’s really structurally sound like, they've been guiding each other on how to fill out the FAFSA and, like, which residence halls, where to get the best fried chicken. [..] And there’s a little bit of stars in their [fans’] eyes when like, one of the good players says this is a good school, but it’s been very supportive.” (P23, 43, Female, Admin)*

Students—both players and workers—were very aware of this program and were even able to name specific fans they had connected with via the Discord channel. They spoke highly of the ability to provide fans with an “inside look” at their university life and esports programs. Many program alumni, according to administrators, remained connected with the recruitment Discord even after graduation, coming back to answer questions and provide college and career advice. In this way, the esports program built their fandom into a force for the university. This subtheme combines player labor with fan labor; while the fans were the ones receiving aid in terms of information and network connections, the goal was then to convert these fans into involved supporters and extend the university's brand impact accordingly.

#### 5.1.2 Legitimization

A more direct form of fan labor appeared in participants’ discussion of how fans helped them legitimize their program to university administrators, resulting in additional funds and resources. As one interviewee put it, “any club trying to get more money from their administration is just gonna run into issues […] If it's not a sport and people aren't coming to events and people aren't paying [for] tickets […], it's not that important for them” (P14, 24, Male, Admin). Having a fan following helped programs overcome these issues. Not only could administrators point to recruitment programs, like those described above, but players and student media workers carefully tracked fan engagement levels to prove that they were reaching audiences. One student broadcaster described:

*“The Twitch channel is everything for the success of the program and having fans on our Twitch channel is going to mean so much. Because it’s also a number you can point to, you can say X amount of people are watching whenever we're streaming, X amount of people are following, which means they're going to get a notification as we go live. And that is a huge step in all the future stuff like funding and whatnot.” P10, 20, Male, Student Worker*

Fans were essential to programs in how they could prove to university administrators that esports mattered, met university goals, and therefore deserved to be supported. In this way, fans unknowingly provided value to collegiate esports programs, helping them gain resources and funding. This can be seen as both a form of fan labor, in how fans produce audiences for events and Twitch streams, but also as a form of audience commodity, as program viewer numbers or “eyeballs” are exchanged for university funding dollars.

#### 5.1.3 Support for student players

Collegiate esports fans’ third role was perhaps the most obvious—supporting players and teams during practice and competition. Collegiate esports players and broadcasters felt that their fans were “awesome” in how they “cheer them on, they tune in to the streams, and they're genuinely excited, like that level of excitement is just refreshing” (P10, 20, Male, Student Worker). One student player simply described the role of fans as providing “energy, motivation, something to play for” (P13, 21, Male, Player) while another said the presence of fans at competitions “just adds a little bit of rivalry to maybe some school matches, and it just makes it more fun” (P4, 21, Male, Player). Describing livestreaming their teams’ tournaments, one administrator said,

*The students love it when they can report that X number of people watch the tournament. […] Or we can get some publicity in some other kind, either on the radio or publication that says, you know, so many people watch the [college esports] team beat somebody else, and you can watch a replay of the game here. So, the students love it. It, it builds our [university] name a little bit, […] and it’s going to become increasingly more important (P18, 62, Female, Admin)*.

This administrator connected university publicity and players’ excitement about fans, showing how many fan roles are overlapping. Players engaged with fans outside of competitions as well, often having fans serve as opponents during in-house practices and matches. They often seemed pleasantly surprised at the fact that people cared about esports, their teams, and their program overall. In this way, fans added emotional and social value to the experience of being on the collegiate esports team or broadcasting matches.

#### 5.1.4 Growth of the industry

Through all the above roles, collegiate esports fans helped normalize and promote the presence of esports on campus. In doing so, they strengthened the collegiate esports industry as a whole. As one student related, “When I was joining the club, I didn't really feel like anyone really respected what esports was. Or even like, they didn't even understand what esports was. […] But definitely over the past couple years, the perception has just been nothing but positivity” (P3, 20, Male, Player). By legitimizing teams, fans helped ensure universities continued to support their esports programs, while connecting with and supporting players, and spreading word to other potential fans, helped expand numbers of fans, potential players, and potential university recruits. To return to De Kosnik's (10) discussion of fan labor, “the community-building labor of fans endows objects with much of their appeal” (p. 102). Collegiate esports fans help build both the economic value—via legitimization and university funding—and social value—via emotional experiences and social connections—of the collegiate esports industry.

### 5.2 Esports fans vs. ball-and-stick fans

Participants also drew connections between collegiate esports fans and ball-and-stick sports fans, suggesting that the former could help redefine fan/athlete relationships. Several interviewees argued that esports athletes connected more with their fans than traditional athletes; “[College basketball] fans don't get to go play pickup games with the players. Well, that's totally different here. Everybody plays with everybody” (P7, 32, Male, Admin). Students similarly felt it was easier to reach out to varsity esports players than traditional sports players, without fear that the player “would have removed me from the premises and security would have me out” (P25, 21, Female, Student Worker). Because collegiate esports did not have the established cachet of traditional collegiate sports, especially at large Division 1 schools where teams were regularly competing on television, fans could build more casual and personal relationships with players.

Participants also pointed out how histories of branding and sponsorship in professional esports, and the fact that esports were generally not considered “sports” at the collegiate level, allowed them to develop unique sponsorship opportunities. Because esports are regulated differently than traditional sports, they undermine many longstanding restrictions on advertising and promotion during events. One program administrator said,

*I have the only licensed jersey on the entire surface of the University [Name] that you can put logos on. Because you can't do that in athletics. So, you take the power of the brands that our universities represent. And now you're making it touchable, approachable, and functional in that way. It will become self-sustaining and revenue generating really fast.” P28, 40, Male, Admin*

While the ethical and moral implications of greater corporate involvement in university spaces are debatable, businesses already have a strong stake in U.S. educational institutions, especially in terms of merchandising and university promotion. Fans and students are already implicated in many indirect forms of economic exchange, from having to consume specific branded products that have contracts with universities (e.g., Pepsi schools vs. Coca-Cola schools) to being exposed to advertisements in campus sports venues. Collegiate esports sponsorships and promotions, as well as the sale of esports team merchandise, extend fan labor in both indirect (audience commodity) and direct (consumer sales) ways.

Finally, existing college sports affiliations benefited esports in turn. As one industry worker framed it, “[collegiate esports] consumers are already organized. They're already members of a college, and they already have, […] consumer loyalty towards their college” (P14, 24, Male, Admin). Recent efforts to found athletic-conference-based esports leagues, such as the Big 10 Conference's unofficial Big Esports League (56), aim to build off existing collegiate affiliations and rivalries as well. This sharply diverges from professional esports, which has struggled to build geographic affiliations and provides a potential lesson in how the industry could better leverage existing fanbases (addressed further below). One player argued, “people are gonna care a lot more about school pride than they will a random esports event or team that might be better than the college team, but they don't know anything about them. They know stuff about [their preferred university.] They know stuff about [their university rival]” (P6, 22, Male, Player). Another related how a family friend, who knew nothing about esports, would view his matches simply to cheer for their shared university; “he had no idea what *Hearthstone* was, but he would tune on and watch us every time and it was a really cool interaction [..] and it was just so, like, wholesome” (P11, 22, Male, Player).

Some participants even mentioned seeing well-known traditional sports alumni tweet about their school's new esports programs; “They tweeted the jersey, the [school] esports team's jerseys. They retweeted them and said, “Man, I wish, I'm so happy these weren't here when I was part of you. Oh, because I would have never left the program” (P9, 22, Male, Student Worker). The connections between college esports and traditional sports, although often unofficial, manifested in several different ways, drawing additional fan attention. This creates what one admin called “goldmine opportunities” to build off existing affiliations and rivalries. Describing his program, he said, “In this part of the country, there are shops built on “a house divided” and [competing local university colors], there are people that are so fanatic. […] It's another way of tapping into preexisting cultures of our campus and our brands” (P28, 40, Male, Admin). Existing sports fandoms became fodder for esports fandoms, again bringing attention and value back to the university, the sports and esports programs, and their sponsors.

### 5.3 Challenges to collegiate esports fandom

Although fans easily fit into existing university, brand, and sponsorship structures in many ways, interviewees did recognize a few challenges to building robust, valuable collegiate esports fandoms. First, our respondents recognized that esports fans were limited in number, understudied, and underutilized—what one director called a reservoir of “unmet potential” (P23, 43, Female, Admin). This was also evident in some player interviews. When asked about fans, they made statements like, “my family is pretty much my fans, so I don't know what it would really be like to have fans besides them” (P24, 19, Female, Player) or “I'd say that like the closest thing called to a fan are my friends tuning in” (P2, 19, Male, Player). One player bluntly said they had no fans “other than our parents right now” (P11, 22, Male, Player). The student broadcasters we interviewed felt their programs had more than just parent-fans, but even they closely tracked their number of Twitch subscribers and tournament viewers, celebrating when over 50 people tuned in for the first time, and then again when they broke 200 Twitch channel subscribers. These are low numbers in the grand scheme of livestreaming and esports, highlighting the embryonic stage in which overall collegiate esports fandom exists. Student workers devoted a great deal of time and energy building and promoting their community rather than managing existing fans—“20% is relating to people and like making sure that everybody's like fine and nothing crazy is happening inside. And then definitely 80% is reaching out to people” (P17, 20, Female, Student Worker).

An additional issue occurred when new fans had to be onboarded to esports. Although existing university affiliations helped bring in potential esports fans, not all competitive game titles are easily understandable to rookie viewers. “Viewership experience is […] something overlooked, actually, that's a huge indicator of if people can actually watch your game or not, if it can become a successful esport” (P14, 24, Male, Admin). One player's mother, for instance, regularly watched matches, but was never sure who was winning. He said, “My mom is also my number one supporter, but […] she said it was the most stressful thing in the world watching those matches because she'd be like, oh, frick, they're gonna lose. And then two turns later, we'd win” (P11, 22, Male, Player). This suggests programs need to develop better engagement strategies moving forward if they want to retain, rather than just recruit, fans for the program. The student mentioned above, for example, found that their mother's game knowledge improved over time thanks to the shoutcasters on their stream, who provided understandable information about the game. Another participant, an administrator, found that having expert players involved in events helped clarify things for rookie attendees. Describing a 700+ person community event their esports program hosted, he said,

*“Probably 75% of the people were like, adults, families, just people who just want to like come out and support things, and they had no idea what the hell was going on in the game. Like no idea. But they knew, they kind of like followed the 25% of the people. […] And like, I was kind of like, “Okay, this can work! Like this, this can be, it can be a lot more.” (P19, 29, Male, Admin)*

Fans are enthusiastic, but because esports is newer and less pervasive than traditional sports, additional work is needed to make the rules and structures of popular games understandable. Players and programs are also somewhat beholden to game publishers here; if publishers make overly complex, difficult-to-understand games, it will be challenging to build a robust, non-expert fandom.

Finally, participants saw game culture's historical insularity and toxicity as a potential limit to fan engagement, expressing concern about how new, non-endemic fans might react to the negativity common to esports spaces. As previous research has addressed, gaming and esports culture is full of trash-talk (57), which can often involve exclusionary sexist, racist, or homophobic language (58–60). Discussing professional esports streaming and fandom, a player participant argued that they found streaming culture to be very harsh on players, “I feel like people who aren't playing the game but are watching someone else play the game tend to be more unforgiving of mistakes, or just like statements or anything that the streamer is doing” (P24, 19, Female, Player). Another participant expressed concern that “online speech has kind of, like, questionable ways of receiving punishment” (P25, 21, Female, Student Worker). These participants and others expressed some concern that the norms of gaming and professional esports may cross over into collegiate esports in ways that would make the space unwelcoming, especially for players who don't fit the masculinized norms of many esports cultures (40, 61).

In addition to potentially borrowing toxic norms from gaming culture, “[Collegiate esports fandom] also allows people to be petty in a new, different way. Because it's like, you know, crap all over [competing school]” (P28, 40, Male, Admin). In other words, esports could both take advantage of and worsen inter-college rivalries. At least a few student players related incidents where trash-talk directed at them during a tournament took the form of insults against their school. “It was done by a small group of people, but nonetheless, they were there. […] And the saddest thing to me, was how our community felt, watching that [opposing players] insult our school” (P13, 21, Male, Player). Fan abuse is an existing, and under addressed, issue in traditional collegiate athletics (62) that merits additional attention in esports spaces as well.

## 6 Discussion

Viewing the above through the lens of fan labor, collegiate esports players, student workers, and administrators all thought fans contributed value to their programs. Many participants felt that cultivating a fanbase could: help justify programs to university administrators, support esports teams financially via attendance at events, online viewing, and merchandise purchases, and even help legitimize programs within broader sports and university institutions. Perceptions of collegiate esports fans therefore closely resemble the roles they currently play in professional esports spaces, where they contribute direct and indirect economic value to teams, games, and the industry (35–38). Fans, at both the collegiate and professional level, also build social and affective ties between participants, helping increase fan and player investment in teams and matches (32, 40). Given the rapid, but still early, growth of collegiate esports programs, fan labor is particularly essential. Throughout our dataset, participants emphasize the challenges of convincing university administrators to take esports seriously, and the importance of fan engagement to these efforts. Fans also provided support systems for players and administrators alike, or benchmarks for mapping personal and program achievements. Therefore, while many media industries rely heavily on fan labor, it takes perhaps an even more central role in collegiate esports spaces.

Our findings also reflect many themes evident in traditional collegiate athletics research. Collegiate athletics programs can be an aid in student recruitment and retention (44); so too can collegiate esports programs, albeit likely on a smaller level and for a more targeted set of fans. Administrators and students enthusiastically described outreach to potential fan recruits and extension of the university brand as a benefit of their esports programs. Student participants, like fans, were therefore also implicated into forms of labor, as their self-promotion and community-building helped draw attention to the university and onboard new applicants. Interviewees positioned these patterns as mutually beneficial, trading student labor for stronger programs and communities, which subsequently drew the interest of fans and allowed them to connect with one another, build an affiliation with the university community, and promote the university brand. Participants also argued that collegiate esports offered novel opportunities to connect with players, providing an accessibility that they felt traditional college sports lacked. Thus, they believed there was strong potential for future esports fandom expansion.

We expect that both collegiate and professional esports will continue to rely heavily on fans in the future. The collegiate model described above, in which esports teams draw on existing school affiliations to build broader fandoms, may also become a model for professional esports. Although the professional esports industry is highly valued, individual teams often struggle to achieve sustainable profitability (63). Replicating traditional or collegiate sports’ broader affiliation networks may be one means to solve this problem. We should be clear—we are not urging collegiate programs to worry about or aim for profitability, nor do we see relying on fans as a panacea for the industry. We bring these points up to emphasize how programs are *already* incorporating fan labor into their business models, making it imperative to understand how this is occurring and what it means, especially given the chance these practices will permeate out to the wider esports industry. Collegiate esports fandom, and associated labor via local networks, introduces regional and location-based elements that are usually absent from esports, which could present both new community-building opportunities but also new chances for audiences and fans to be packaged as audience commodities for advertisers.

Although we did not directly address esports fans in this dataset, a limitation described below, collegiate esports participants implied that their programs offered many benefits to fans, such as strong connections with players and community building opportunities. These also connect to an extent with existing studies of esports and traditional sports fans, which rank social connections and relatedness needs (33, 42) and campus trust and social capital (6, 48) as key benefits of fandom. It's important to note, however, that interview participants often *assumed* fans would garner these benefits, rather than showing how their programs directly supported such outcomes. Participants primarily focused on what fans brought to the programs in terms of value, rather than vice versa. Even answering potential students’ questions about the university, which aided those individuals, was part and parcel of extending the university's brand power and increasing enrollment numbers. Thus, benefits to fans themselves (information, social connections, etc.) were secondary to how fan labor helped establish and legitimize programs. Further research with fans is needed to elaborate which, if any, benefits they see in their fandom.

Finally, some participants mentioned challenges to building robust, welcoming fandoms, such as the fledgling status of collegiate esports. Although collegiate esports programs have expanded rapidly, they have not necessarily “cracked the code” on drawing in non-game-playing students, alumni, or community members. In part, this is because esports can be confusing to unfamiliar viewers; unless a spectator is themselves a player, watching a *League of Legends* or *Overwatch* match may not make sense. Programs need to rely heavily on shoutcasters [who are often student workers; (28)] to parse out the action of an esports match in ways that make sense to viewers. Student labor is thus needed to build a foundation for successful fandom. Additionally, game culture's historic toxicity, which is particularly unwelcoming for non-male, LGBTQ+, or POC players and fans, presents another obstacle. These are not insurmountable issues; traditional sports, for instance, has often been toxic (62) and unwelcoming to many potential fans (64, 65), issues that are still being addressed across different leagues. Traditional sports can also have confusing rules that need to be explained to new fans, as with esports. Gaming and esports, however, do not yet have the history and institutions sports use to manage these issues. They will need to develop these structures, requiring still more labor from fans and institutions alike, to continue expanding the fanbases on which they already rely. Colleges could, with appropriate scaffolding, be a useful location in which to advance efforts towards inclusivity (52), but it will be important to investigate how these efforts occur and which participants (players, fans, administrators, etc.) are involved in the work of building inclusive spaces and communities.

Games and esports are highly fan-reliant industries, and collegiate esports is thus far following similar trends. Thus, it serves as a useful area in which to investigate and potentially intervene in fan practices and work. Many of the perceived outcomes of collegiate esports fandom rest on the work and engagement of fans themselves as well as of the (generally unpaid) students workers who manage communities and livestreams (28). Thus, we recognize the benefits that fandom offers, but we also remain wary of potential overreliance on free fan and student labor. Moreover, program initiatives will require thoughtful strategies for effective community management and moderation to ensure positive outcomes for programs, players, and spectators.

### 6.1 Limitations

One limitation of this article is that, although it is about fans, data was not collected directly from fans. This is due, in part, to the fact that the broader interview project focuses primarily on collegiate esports programs’ growth and institutionalization. That focus necessitates prioritizing members of programs. We also started data collection during the COVID-19 pandemic in 2020, when both in-person events and collegiate streaming were disrupted by remote learning. It was, in this circumstance, easier to connect with program participants via peer networks rather than to connect with more nebulous groups such as fans. Therefore, while we can speak to how program participants perceive and position esports fans (the research questions for this article), we don't have a full understanding of how fans themselves view their involvement and labor in esports. Future research must add fans voices to the mix to understand their position fully.

Additionally, and unsurprisingly for work on esports (66), our participants were primarily young and male, especially in terms of esports players. Therefore, while they express the perspectives of esports’ dominant groups, we may not see the full range of roles or issues concerning collegiate esports fans. Future work should aim to diversify recruitment to address marginalized players’ and administrators’ perspectives more fully.

## 7 Conclusion

As esports institutionalizes and spreads across U.S. college campuses, it is important to understand how this growth is occurring and what patterns or structures are being normalized in this field. In terms of collegiate esports fans, our study suggests that players, administrators, and student workers celebrate the presence of fans, not only for the social benefits that players and student broadcasters receive from having people watch their matches, but also because fans do essential labor for programs, helping support them, legitimize their existence to university administrators, and grow the collegiate esports environment. Interviewees also suggested that fans could benefit the university, both by extending the university brand, building community ties, and upping recruiting power among young audiences, but also by normalizing new patterns of sponsorship and funding previously barred in traditional collegiate sports. As colleges grapple with changes in name, image, and likeness (NIL) rights for student athletes, for instance, esports and esports fans may provide models upon which to draw in the sports environment (67).

At the same time, programs should be cautious to avoid exploiting free fan labor. While our participants suggested that fans received benefits from their interaction with collegiate esports programs, such as fun, community ties, and inside information about a university, additional research with fans is needed to provide evidence for these claims. Otherwise, there is a risk that programs will increasingly package students and fans as audience commodities for game companies and sponsors without also ensuring positive social or educational outcomes. Moreover, current patterns in collegiate esports program development show a strong reliance on student labor to connect with fans, moderate Discord servers, create broadcasts, and explain the progress of matches (28). This often-unpaid work helps build the fandoms that programs rely on to legitimize themselves to university administrators, but it also may undervalue student laborers. We suggest that programs continue to think critically about the costs and benefits players, student workers, and fans incur from their participation in collegiate esports programs and to support those whose labor allows programs to function. What emerges at least in these still relatively early days of formal collegiate esports is a picture of an at once optimistic and mutually beneficial relationship between fans and teams. Ultimately, we advocate for the careful support of this symbiosis particularly as programs require more labor, investment, and fans to grow.

## Data Availability

The datasets presented in this article are not readily available because of potential identifying information, the terms of our Institutional Review Board approval, and the fact that constructivist research does not aim for replication. Requests to access the datasets should be directed to Amanda Cote, acote@msu.edu.

## Footnotes

^1^Fan labor and free labor are deeply intertwined with several other theories such as playbor [the increasing intertwining of work and play; (19)] and immaterial labor [“labor that produces an immaterial good such as a service, a cultural product, knowledge, or communication,” (20), 290]. We focus on fan labor both for the sake of brevity and because it accounts for the range of possible fan behaviors, from buying or creating physical goods to moderating an online Twitch chat, and their varied contributions to the broader esports industry.
